# Students’ Society

**Published:** 1890-01

**Authors:** 


### Students' Society.

The students of the New York College of Dentistry have a regularly organized society for mutual improvement. The advantages of such associations are beyond question, and in many ways will result in good to those engaged. They have also established a journal, bearing the title "*The Record*," published monthly. These will be a means of valuable education and culture to the classes.

All dental colleges should encourage their classes to organize and carry on societies, and in this way derive a benefit that can not be supplemented by anything in the ordinary college course.

